# Letter to the Editor regarding the article: "identifying pre-hospital factors associated with outcome for major trauma patients in a regional trauma network: an exploratory study"

**DOI:** 10.1186/s13049-017-0454-1

**Published:** 2017-11-25

**Authors:** Charlie A. Sewalt, Eveline J. A. Wiegers, Esmee Venema, Hester F. Lingsma

**Affiliations:** Center for Medical Decision Making, Department of Public Health, Erasmus University Medical Center, P.O. Box 2040, 3000 CA Rotterdam, The Netherlands

**Keywords:** Predictive factors, Prediction modelling, Methodology

## Abstract

The aim of this Letter to the Editor was to report some methodological shortcomings in a recently published article. Issues regarding missing values and overfitting are mentioned. First, Complete Case (CC) analysis was used instead of an imputation method. Second, there was a high chance of overfitting and lack of model validation. In conclusion, the results of this study should be interpret with caution and further research is necessary.

With great interest we read the study by Thompson et al. [1] where they identified pre-hospital factors associated with major trauma outcomes. This study showed that Glasgow Coma Score (GCS), Respiration Rate (RR) and Age are potential predictive triggers for direct transport to a Major Trauma Center (MTC). This is an interesting finding, which might help in the challenging decision which patients will benefit from treatment in MTCs. However, some methodological issues should be taken into consideration.

First, the authors used ‘listwise’ exclusion, also known as complete case (CC) analysis, to handle their missing data. This resulted in excluding almost 45% of their entire sample (462 out of 1033 casualties). Obviously, this leads to less efficiency and possibly bias [2, 3]. In dealing with missing data, the CC analysis could be biased when Missing at Random (MAR) on the outcome variable is present [2–4], for example when GCS is missing in patients with high GCS who have a high probability to die. Thus imputation methods should have been considered. This could certainly increase efficiency and potentially reduce bias dependent on the mechanism of missing data [2–4].

Second, the authors stated that their model including GCS, RR and Age correctly predicted 97.4% of the casualties. This high prediction rate could be the result of overfitting and might not be generalizable to the population [3]. Three strategies could be followed to avoid overfitting. First, the use of a more liberal *p*-value than 0.050 and preselection of variables based on clinical knowledge could have decreased the chance of testimation bias and overestimation of the effect of the selected predictors, especially with few events [3, 5]. Two other important steps in prediction modelling are internal and external validation [3, 6]. Internal validation is about the stability of the selected predictors and the quality of the predictions in the underlying population [3, 6]. External validation is about the generalizability of the predictors and predictions in comparable populations [3, 6]. Unfortunately none of these strategies to decrease overfitting and increase the quality of the prediction models have been used and therefore overfitting is likely in this study.

In conclusion, results of this study should be interpret with caution and further research is necessary to estimate the predictive ability of pre-hospital factors with special emphasis on model validity and overfitting.

## Abbreviations

CC: Complete case
GCS: Glasgow coma score
MAR: Missing at random
MTC: Major trauma center
RR: Respiration rate
